# Evolutionary Couplings and Molecular Dynamic Simulations Highlight Details of GPCRs Heterodimers’ Interfaces

**DOI:** 10.3390/molecules28041838

**Published:** 2023-02-15

**Authors:** Karim Widad Temgbet Nchourupouo, Jules Nde, Yannick Joel Wadop Ngouongo, Serge Sylvain Zekeng, Bernard Fongang

**Affiliations:** 1 Laboratory of Mechanics, Materials, and Structures, Department of Physics, Faculty of Science, University of Yaoundé I, Yaoundé P.O. Box 812, Cameroon; 2 Department of Physics, University of Washington Seattle, Seattle, WA 98105, USA; 3 Glenn Biggs Institute for Alzheimer’s & Neurodegenerative Diseases, University of Texas Health Science Center at San Antonio, San Antonio, TX 78229, USA; 4 Department of Biochemistry and Structural Biology, University of Texas Health Science Center at San Antonio, San Antonio, TX 78229, USA; 5 Department of Population Health Sciences, University of Texas Health Science Center at San Antonio, San Antonio, TX 78229, USA

**Keywords:** direct coupling analysis, coevolution, G-protein coupled receptors, protein-protein interactions, molecular dynamic simulations

## Abstract

A growing body of evidence suggests that only a few amino acids (“hot-spots”) at the interface contribute most of the binding energy in transient protein-protein interactions. However, experimental protocols to identify these hot-spots are highly labor-intensive and expensive. Computational methods, including evolutionary couplings, have been proposed to predict the hot-spots, but they generally fail to provide details of the interacting amino acids. Here we showed that unbiased evolutionary methods followed by biased molecular dynamic simulations could achieve this goal and reveal critical elements of protein complexes. We applied the methodology to selected G-protein coupled receptors (GPCRs), known for their therapeutic properties. We used the structure-prior-assisted direct coupling analysis (SP-DCA) to predict the binding interfaces of A2aR/D2R, CB1R/D2R, A2aR/CB1R, 5HT2AR/D2R, and 5-HT2AR/mGluR2 receptor heterodimers, which all agreed with published data. In order to highlight details of the interactions, we performed molecular dynamic (MD) simulations using the newly developed AWSEM energy model. We found that these receptors interact primarily through critical residues at the C and N terminal domains and the third intracellular loop (ICL3). The MD simulations showed that these residues are energetically necessary for dimerization and revealed their native conformational state. We subsequently applied the methodology to the 5-HT2AR/5-HTR4R heterodimer, given its implication in drug addiction and neurodegenerative pathologies such as Alzheimer’s disease (AD). Further, the SP-DCA analysis showed that 5-HT2AR and 5-HTR4R heterodimerize through the C-terminal domain of 5-HT2AR and ICL3 of 5-HT4R. However, elucidating the details of GPCR interactions would accelerate the discovery of druggable sites and improve our knowledge of the etiology of common diseases, including AD.

## 1. Introduction

Protein-protein interactions (PPIs) are crucial in many biological processes that regulate cellular activities. Therefore, understanding how proteins interact and determining the binding interfaces is critical to drug discovery [1]. Indeed, heteroreceptor complexes, including G-protein coupled receptors (GPCRs), and their allosteric receptor-receptor interactions are now recognized as novel drug targets for many diseases, including neurological disorders [2,3,4,5,6,7]. The GPCRs form hetero-oligomers endowed with various biochemical and pharmacological properties distinct from the corresponding monomers [8]. Tremendous progress has been made in describing GPCRs oligomerization. However, identifying residues driving the interaction is still experimentally and computationally challenging. Several computational techniques have been developed to predict native contacts in protein complexes, including the Direct Coupling Analysis (DCA), which relies on residue coevolution [4,9,10,11,12,13]. The DCA exploits the fact that proteins’ structure and function are conserved at the molecular level, whereas amino-acid sequences may vary between homologous proteins [14,15,16,17]. Historically, the DCA algorithm has been used to accurately predict the 3D structure and coevolving residues of small protein dimers (<100 amino acids). However, the DCA and other computational methods based on residue coevolution generate many false positives (FPs) for larger proteins. Thus, additional postprocessing steps are generally needed to improve their accuracy.

The prediction of coevolving residues in GPCRs oligomers is even more challenging because of their multi-domain structure, which requires a large number of homologous sequences to generate enough statistical power to observe significant co-mutations. To reduce FPs, we have introduced a downstream processing method that convolves DCA signals and structural properties of interacting proteins to filter out FPs [4,18]. Our approach, the Structure Prior-assisted DCA (SP-DCA), uses a Gaussian convolution algorithm with a kernel constructed on the structural information (secondary structure, including topology domains, buried/exposed residues, etc.) of both proteins to predict the binding interfaces of proteins. For example, it was used to predict the interaction between serotonin receptors 5-HT2AR and 5-HT2CR [4] and extended to the RAC1/IMPDH2 [18] interaction, which was later confirmed experimentally. Furthermore, as the SP-DCA approach integrates protein topology domains and evolutionary-coupling scores from DCA, it increases the sensitivity and specificity of predicting the native contacts. We, therefore, hypothesize that SP-DCA can be used to predict interactions for any GPCR heterodimers and other protein complexes.

Although SP-DCA can efficiently predict protein complexes’ binding interfaces, the method cannot provide structural, functional, or mechanistic details of the interacting peptides. Such information can be obtained through molecular dynamics (MD) simulations, an in-silico approach generally used to determine protein structure, dynamics, and functions. However, given their multi-domain structure and size, all-atom MD simulation is challenging to implement for GPCRs.

In the present study, we have developed a novel strategy to determine mechanistic details of PPIs hot-spots that combines unbiased identification of the most likely interacting peptides using SP-DCA, followed by biased MD simulation. We have applied the method to predict the binding interfaces of A2aR/D2R, CB1R/D2R, A2aR/CB1R, 5HT2AR/D2R, and 5-HT2AR/mGluR2 receptor heterodimers and demonstrate its ability to reproduce experimental results. Finally, we have used the two-step procedure to show that 5-HT2AR and 5-HTR4R form a heterodimer and identified their interface and mechanistic details.

## 2. Results and Discussion

### 2.1. SP-DCA Accurately Predicts GPCRs Heterodimers’ Binding Interfaces

**Figure 1:**
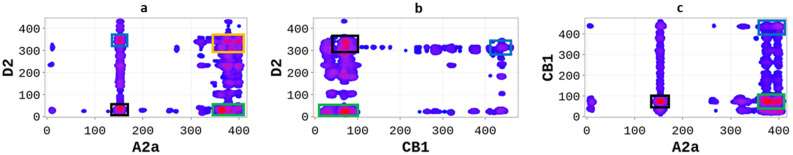
SP-DCA map using a total number of interacting aa, *l* = 21 with optimized parameters 𝑎 = 𝑏 = 0.001 for A2aR/D2R (**a**), A2aR/CB1R (**b**), and CB1R/D2R (**c**). Amino acids are gradient color-coded: no interaction (white areas), medium interaction (blue), and highest probability of interaction (red spots). The rectangles delimit each heterodimer’s most likely interacting peptides (see text).

GPCRs heterodimers were selected based on their pharmacological relevance and the availability of structural data to compare our predictions. The A2aR/D2R heterodimer integrates the signals of two different neurotransmitter systems, allowing adenosine to control the dopaminergic effects of the neurotransmission in an antagonistic way [19,20]. Previous research has shown that A2aR/D2R operates as an integrator of two different neurotransmitters in the striatal column modulus [20,21]. Therefore, a detailed annotation of the heterodimer’s hot-spots could allow the design of therapeutic peptides targeting protein-protein interactions. We applied the SP-DCA methodology to predict the binding interfaces of A2aR/D2R, adjusting the parameters (normalized variances a=b=0.001 and an average length of interacting peptides l=21) as described in Table 1. As shown in Figure 1a, the SP-DCA method highlighted A2aR/D2R hot-spots with the most likely binding interfaces being: (1) the C-terminal domain of A2aR (AA: R291-S412) and the N-terminal domain of D2R (AA: M1-Y37) represented by the green rectangle; (2) the extracellular loop 2 (ECL2, AA: N144-E173) of A2aR and the intracellular loop 3 of D2R (ICL3, AA: I214-Q373) represented by the blue rectangle; (3) the extracellular loop 2 (ECL2, AA: N144-E173) of A2aR and the N-terminal domain of D2R (AA: M1-Y37) represented by the black rectangle.

The SP-DCA map of A2aR/D2R highlights the most likely interacting peptides, all of which were previously shown to be the hot-spots of the heterodimer. Borroto-Escuela et al. used the bioluminescence resonance energy transfer (BRET) technique to show that A2aR and D2R interact mainly through the C-terminal of A2aR and the N-terminal of D2R; the C-terminal of A2aR and ICL3 of D2R; and the TM4 of A2aR and TM4 of D2R; the N-terminal of A2aR and TM5 of D2R [21]. Although coevolutionary-based methods generally performed poorly in transmembrane domains (TM) due to high residue conservation, it is worth noting that the SP-DCA algorithm accurately predicted all non-TM interactions and unveiled new hot-spots.

Similar to A2aR/D2R, functional interactions have been shown to exist between the cannabinoid receptor (CB1R) and the dopamine receptor (D2R) [22]. Moreover, it has been demonstrated that activation of the D2R receptor enhances the release of the endocannabinoid striatal, thus promoting the inhibition of presynaptic glutamatergic neurotransmission mediated by CB1R [23]. However, whether the resulting complex comes from direct or indirect coupling is unclear. We used the SP-DCA method to predict the most likely interacting peptides of the CB1R/D2R heterodimer with the parameters described in Table 1. The SP-DCA map of CB1R/D2R (Figure 1b) shows that CB1R and D2R interact mainly through the (1) N-terminal of CB1R (AA: M1-Q116) and N-terminal of D2R (AA: M1-Y37) depicted by the green rectangle; (2) the C-terminal of CB1R (AA: R400-L472) and ICL3 of D2R (AA: I214-Q373) represented by the blue rectangle; (3) the N-terminal of CB1R (AA: M1-Q116) and ICL3 of D2R (AA: I214-Q373) represented by the black rectangle. Several approaches have been previously used to study CB1R/D2R heterodimerization. For example, Agnati et al. [24] used the fluorescence resonance energy transfer (FRET) technique and the co-immunoprecipitation (Co-IP) assay to establish the existence of the CB1R/D2R complex in co-transfected HEK-293 cells. They subsequently showed that CB1R and D2R interact through the domains of the C-terminal of CB1R (AA: R400-L472) and ICL3 of D2R (AA: I214-Q373), which aligns with our predictions (I214-Q373).

**Table 1:** Summary of the SP-DCA predicted and previous evidence of interactions between selected GPCRs complexes and peptides selected for molecular dynamic simulations.

Complex	cMSA Length	Reported Evidence	Predicted Interactions (SP-DCA)	Peptides Selected for MD
A2aR/ D2R	193	C-Ter/ICL3 [21] C-Ter/N-Ter [21] TM4/TM4 [21] N- Ter/TM5 [21]	C-Ter/N-Ter ECL2/N-Ter ECL2/ICL3	A2aR: 61 AA, CYS245-SER305 D2R: 66 AA, ASN35-TRP100
CB1R/ D2R	404	C-Ter/ICL3 [24] TM4/TM4 [24]	N-Ter/N-Ter C-Ter/ICL3 N-Ter/ICL3	CB1R: 107 AA, ALA19-LEU125 D2R: 223 AA, ARG150-THR372
A2aR/ CB1R	303	C-Ter/C-Ter [24] TM4/TM4 [24] TM4/TM4 [24]	C-Ter/N-Ter ECL2/N-Ter C-Ter/C-Ter	A2aR: 101 AA, ILE100-ILE200 CB1R: 107 AA, ALA19-LEU125
5-HT2AR/ D2R	518	C-Ter/ICL3 [25,26,27]	N-ter/N-ter C-ter/N-ter C-Ter/ICL3 N-Ter/ICL3	D2R: 79 AA, THR144-ARG222 5-HT2AR: 89 AA, GLN313-GLU401
5-HT2AR/ mGluR2	626	TM5/TM4 [28] TM5/TM4 [28] TM4/TM5 [28] TM4/TM5 [28]	N-Ter/N-Ter C-Ter/N-Ter C-Ter/C-Ter N-Ter/C-Ter	5-HT2A: 117 AA, TRP200-SER316 mGluR2: 77 AA, LYS24-GLU100
5-HT2AR/ 5-HTR4R	515		N-ter/ICL3 C-ter/ICL3 N-ter/C-ter C-ter/C-ter	5-HT2AR: 33 AA, THR69-LEU101 5-HTR4R: 154 AA, ARG150-303ASP

Next, we used the SP-DCA with the parameters outlined in Table 1 to predict the binding interfaces of the A2aR/CB1R, which have been previously shown to form a functional heterodimer [24]. As shown in Figure 1c, A2aR and CB1R interact through the (1) C-terminal domain of A2aR (AA: R291-S412) and the N-terminal domain of CB1R (AA: M1-Q116) represented by the green rectangle; (2) the C-terminal of A2aR (AA: R291-S412) and the C-terminal of CB1R (AA: R400-L472) represented by the blue rectangle; (3) the ECL2 of A2aR (AA: N144-E173) and N-terminal of CB1R (AA: M1-Q116) represented by the black rectangle. These predictions align with experimental results by Agnati et al. [24], showing that the two proteins interact at the C-terminal of A2aR and at the C-terminal of CB1R and in the transmembrane domains TM4 of A2aR and TM4 of CB1R.

**Figure 2:**
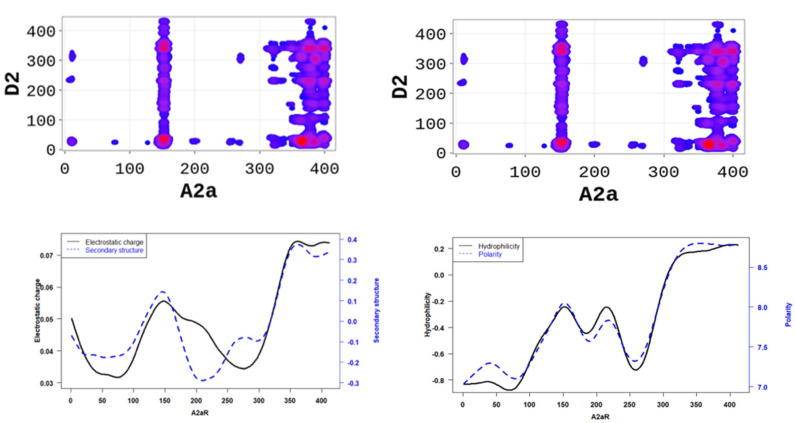
A2aR/D2R dimerization is driven by the secondary structure A2aR and its electrostatic charges. On average, electrostatic charges and secondary structure similarity score (dominated by alpha-helix conformation) of amino acids are higher at the predicted hot-spots (**left panel**). We also observed that predicted interacting residues have high hydrophobicity and polarity scores (**right panel**).

**Figure 3:**
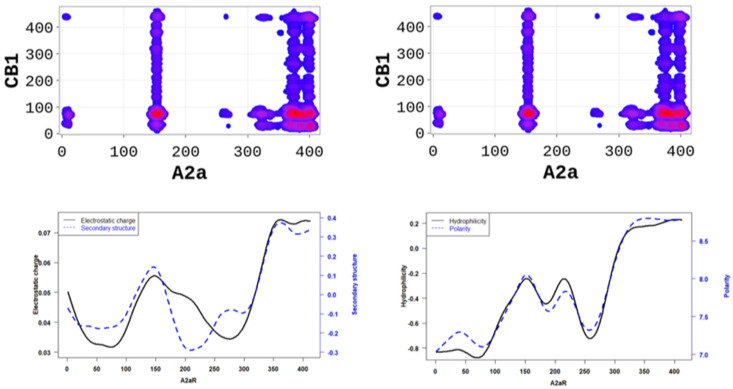
A2aR/CB1R dimerization is driven by the polarity of A2aR and its hydrophilicity. On average, polarity and hydrophilicity (**left panel**) of amino-acids are higher at the predicted hot-spots. Additionally, on average, electrostatic charges (**right panel**) of these amino-acids are higher at the predicted hot-spots and their secondary structures are dominated by alpha-helix conformation.

In order to better understand the nature of the interactions, we estimated the physicochemical properties of A2aR driving the heterodimerization with D2R by averaging known property metrics on the sequence length (Figure 2). We found that A2aR/D2R hot-spots correspond to regions with higher electrostatic charge and a higher secondary structure similarity score. In addition to the secondary structure folds and electrostatic properties, the dimerization of A2aR is also driven by the minor contributions of polarity and hydrophilicity, as shown in Figure 2. Similarly, the binding of A2aR to CB1R is also driven by electrostatic, polarity, hydrophilicity, and secondary structure interactions, as shown in Figure 3.

### 2.2. SP-DCA Highlights Details of the Differential Heterodimerization of 5-HT2AR

Heterodimerization of serotonin receptors opened up a new avenue for differential regulation of signaling by enhancing or inhibiting the original pathways activated by the cognate homodimers [25,26,27]. For example, co-activation of the 5-HT2AR receptor co-expressed with the µ-opioid peptide (MOP) receptor in HEK293 cells results in the MOP receptor agonist morphine being able to induce down-regulation of the MOP receptor [26]. Although very important, the serotonin receptors are less exploited than dopamine receptors as therapeutic targets. Serotonin (5-HT2AR), involved in learning and cognition, is widely distributed in the central nervous system. Preclinical studies showed that abnormal 5-HT2AR receptor activity is involved in psychiatric disorders, including depression and drug addiction [25].

**Figure 4:**
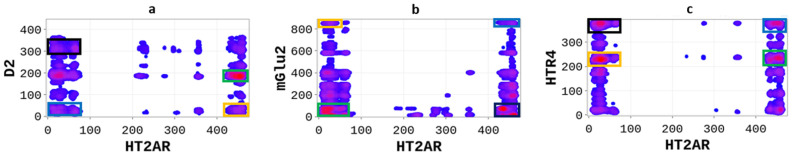
Differential heterodimerization of 5-HT2AR/D2R (**a**), 5-HT2AR/mGluR2 (**b**), and 5-HT2AR/5-HTR4R (**c**). The SP-DCA maps were obtained as described in the text using a total number of interacting aa, 𝑙 = 21 with optimized parameters 𝑎 = 𝑏 = 0.001. Amino acids are gradient color-coded: no interaction (white areas), medium interaction (blue), and highest probability of interaction (red spots). The rectangles delimit each heterodimer’s most likely interacting peptides (see text).

We sought to explore how the serotonin 2A (5-HT2AR) receptor differentially heterodimerizes with other GPCRs, including the dopamine receptor 2 (*D2R*), the glutamate receptor 2 (*mGluR2*), and the serotonin receptor 4 (*5-HT4R*). Therefore, we performed SP-DCA analyses for each heterodimer and explored the different physicochemical properties driving the interactions. The SP-DCA maps of 5-HT2AR/D2R, 5-HT2AR/mGluR2, and 5-HT2AR/5-HT4R are shown in Figure 4 with an indication of the most likely interacting peptides.

**Figure 5:**
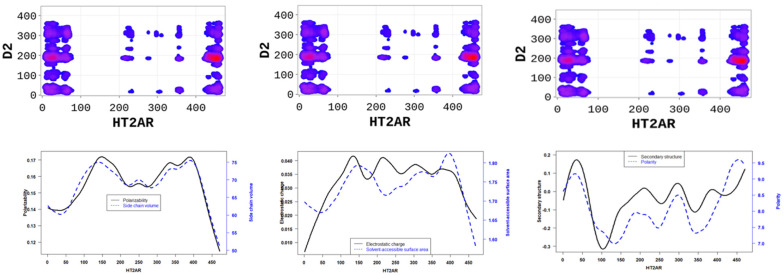
5-HT2AR/D2R dimerization is mainly is driven by (1) the side chain volume and polarizability scores (**left panel**); (2) the electrostatic charge and solvent accessibility scores (**middle panel**); and (3) the secondary structure similarity (dominated by alpha-helix conformation) and polarity scores (**right panel**) of 5-HT2AR residues.

The SP-DCA analysis of the 5-HT2AR/D2R heterodimer (Figure 4a) indicates that both receptors interact through (1) the C-terminal domain of 5-HT2AR and the N-terminal domain of D2R (AA: M1-Y37); (2) the N-terminal domain of 5-HT2AR (AA: M1-N75) and the N-terminal domain of D2R (AA: M1-Y37) represented by the blue rectangle; (3) the N-terminal domain of 5-HT2AR (AA: M1-N75) and ICL3 of D2R (AA: I214-Q373) represented by the black rectangle; (4) the C-terminal domain of 5-HT2AR (AA: K385-V471) and the ICL3 of D2R (AA: I214-Q373) represented by the orange rectangle. The 5-HT2AR and D2R have been previously shown to interact through the C-terminal of the 5-HT2AR and the ICL3 of the D2R using the FRET method [27]. In addition to the binding interface, we found that the dimerization of the 5-HT2AR and D2R, as predicted by the SP-DCA, is driven by the 5-HT2AR/D2R’s several physicochemical properties, including hydrophobicity, polarity, electrostatics, polarizability, side chain volume, solvent-accessible surface area, and the predicted secondary structure folding of residues (Figure 5).

The interaction between the 5-HT2AR and mGluR2 has been extensively studied both experimentally and theoretically [28,29] over the last decades. However, these studies, achieved through FRET and molecular dynamics simulations, suggested that 5-HT2AR and mGluR2 could only heterodimerize at their transmembrane domains. Our SP-DCA analysis (Figure 3b) predicts additional hot-spots at the (1) N-terminal of 5-HT2AR (AA: M1-N75) and N-terminal of mGluR2 (AA: I19-I567) indicated by the green rectangle; (2) C-terminal domain of 5-HT2AR (AA: K385-V471) and the C-terminal domain of mGluR2 (AA: L820-G872) represented by the blue rectangle; (3) the C-terminal of 5-HT2AR (AA: K385-V471) and the N-terminal of mGluR2 (AA: I19-I567) represented by the black rectangle; (4) the N-terminal domain of 5-HT2AR (AA: M1-N75) and the C-terminal of mGluR2 (AA: L820-G872) represented by the orange rectangle.

Additionally, we applied the SP-DCA method to understand the 5-HT2AR and 5-HTR4R heterodimerization mechanisms, for which no structural data is available. However, the 5-HTR4 receptor [30,31] is a human serotonin receptor located in the intestinal tract as well as the central nervous system, that functions in both the peripheral and central nervous systems to modulate the release of multiple neurotransmitters [32]. As a result, figuring out how it interacts with 5-HT2AR is crucial for brain health. We hypothesized that 5-HT2AR and 5-HTR4R would form a functional heterodimer and used the SP-DCA algorithm to predict the most likely interacting peptides. The analysis was performed as previously described, with the total number of the interacting peptides aa=21 and optimized parameters a=b=0.001. As shown in Figure 4c, the 5-HT2AR and 5-HTR4R mainly interact through (1) the C-terminal domain of 5-HT2AR (AA: K385-V471) and the ICL3 of 5-HTR4R (AA: R214-T260) represented by the green rectangle; (2) the C-terminal domain of 5-HT2AR (AA: K385-V471) and the C-terminal of 5-HTR4R (AA: N316-T388) represented by the blue rectangle; (3) the N-terminal domain of 5-HT2AR (AA: M1-N75) and the C-terminal of 5-HTR4R (AA: N316-T388) represented by the black rectangle; (4) the N-terminal domain of 5-HT2AR (AA: M1-N75) and the ICL3 of 5-HTR4R (AA: R214-T260) represented by the orange rectangle.

**Figure 6:**
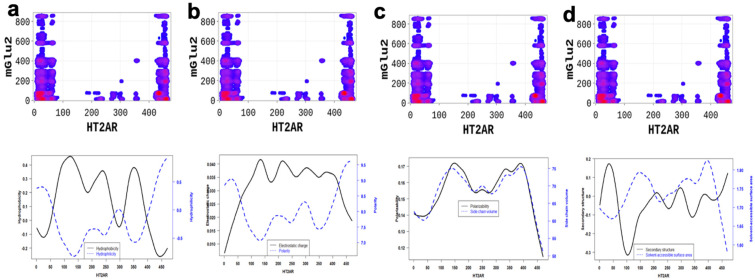
5-HT2AR/mGlu2R dimerization is mainly driven by (**a**) the hydrophilicity, (**b**) the electrostatic charge and polarity, (**c**) the polarizability and side chain, and (**d**) the secondary structure similarity and solvent accessibility properties of the 5-HT2AR amino-acids located at the interfaces. On average, polarity and hydrophilicity properties of amino-acids are higher, however secondary structure, electrostatic charge, polarizability, side chain volume, and solvent-accessible surface area property are lower at the predicted hot-spots and their secondary structures are dominated by alpha-helix conformation.

**Figure 7:**
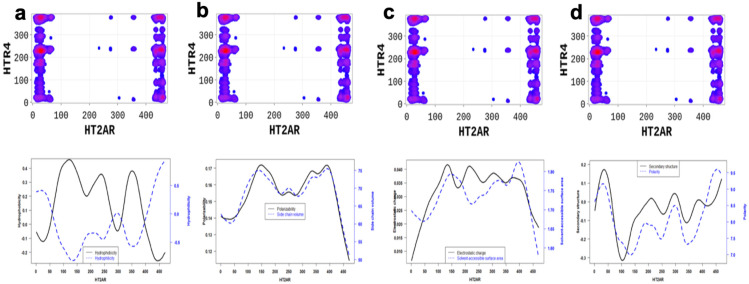
5-HT2AR/5-HTR4R dimerization is highly driven by (**a**) the hydrophilicity, (**b**) the polarizability and side chain, (**c**) the electrostatic charge and polarity, and (**d**) the secondary structure similarity and solvent accessibility properties of the 5-HT2AR amino-acids located at the interfaces. These properties are significantly different at the predicted binding interfaces than non-binding peptides.

Altogether, our results highlight essential residues involved in the dimerization of 5-HT2AR and the physicochemical properties necessary for these interactions. Binding interfaces of 5-HT2AR are peptides with low hydrophobicity, electrostatic charge, polarizability, side chain volume, and solvent-accessible surface area properties, and high polarity, high hydrophilicity, and high secondary structure (Figure 6 and Figure 7).

### 2.3. Molecular Dynamics Simulations Reveal Details of Interacting Peptides

The SP-DCA approach is an unbiased method to predict the binding interfaces of proteins but generally fails to give mechanical and structural details of the interacting peptides. On the other hand, all-atom molecular dynamics (MD) simulation is a powerful method for investigating protein dynamics and protein-protein interactions at atomic levels [31,32,33,34,35]. However, given the high computational cost of all-atom MD, its application to studying the dynamics of large proteins, including GPCRs, is a notoriously tricky task. Thus, to investigate the dynamics of the predicted heterodimers, we used a biased approach in which we selected only the most likely interacting peptides of both proteins for MD simulations. Due to this selection, our results cannot lead to definite conclusions. However, they help identify potential key residues, visualize trajectories along the interaction pathway, and reveal critical features that can be tested experimentally [18].

**Figure 8:**
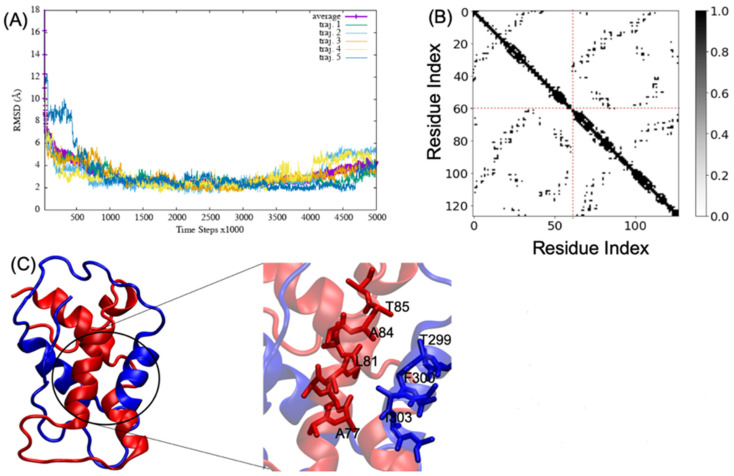
Successful association between the N-terminal domain of D2R and the C-terminal domain of A2aR. (**A**) shows the root mean square deviation (RMSD) over five trajectories and its average; (**B**) indicates the residue contact maps where the dashed lines limit the two monomers; residues 1 to 61 represent the A2AR, and residues 62 through 127 represent D2R; (**C**) shows a snapshot of the complex A2AR (blue) and D2R (red). A few residues at the interacting interface between A2AR and D2R are zoomed out to showcase their interactions. We visualized the structures by using the VMD program.

**Figure 9:**
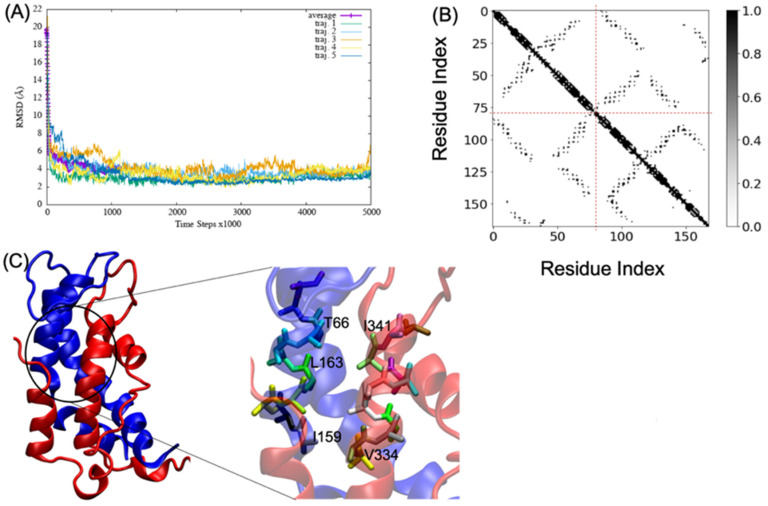
Successful association between the N-terminal domain of D2R and the C-terminal domain of 5-HT2AR. (**A**) shows the root mean square deviation (RMSD) over five trajectories and its average; (**B**) indicates the residue contact maps, the dashed lines limit the two monomers; residues 1 to 79 represent the D2R, and residues 80 through 168 represent 5-HT2AR; (**C**) shows a snapshot of the complex D2R (blue) and 5-HT2AR (red). A few residues at the interacting interface between D2R and 5-HT2AR are zoomed out to showcase their interactions.

**Figure 10:**
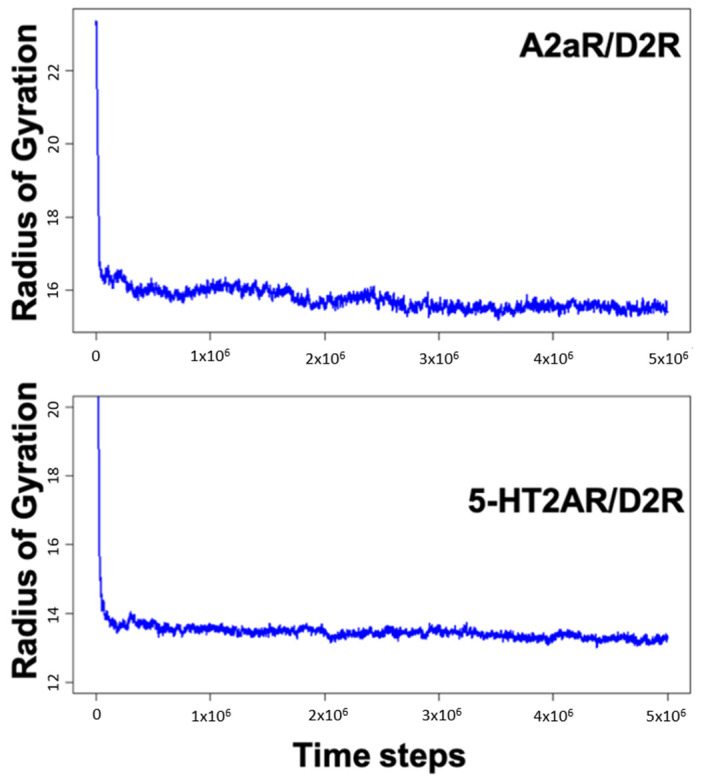
Variations of the radius of gyration as a function of time steps for the C-terminal of A2aR and N-terminal of D2R and then the C-terminal of HT2AR and ICL3 of D2R.

MD simulations of A2aR/D2R and 5-HT2AR/D2R heterodimers using predicted binding interfaces (Table 2) are presented in Figure 1 and Figure 4. For the A2aR/D2R heterodimer, we selected the C-terminal of A2aR and the N-terminal of D2R for MD. This region corresponds to our predicted hot-spots and was previously shown as an essential domain in the complex because it supports allosteric modulation in the interactions between the two proteins [36]. Similarly, we selected the C-terminal of 5-HT2AR and ICL3 of D2R for MD simulations to shed light on the binding mode of these hot-spot regions. The dynamical properties of the two domains were further subjected to trajectory data analysis. The root means square deviation (RMSD) was recorded along the simulation for five trajectories, and its average was computed along the entire simulation (Figure 8A and Figure 9A). The RMSD average in both hot-spots showed stability, with an RMSD value of around 3 Å for the C-terminal of A2aR and the N-terminal of D2R (Figure 8A) and around 4 Å for the C-terminal of 5-HT2AR and ICL3 of D2R (Figure 9A). Technically, all two domains reached equilibrium despite fluctuations along the MD process. The two monomers are initially placed at a distance of 30 Å from each other, which explains the high variations before the encounter around 1,000,000-time steps (Figure 8A and Figure 9A). Furthermore, slight deviations around 450,000-time steps could imply that transient contacts are broken, allowing the most stable contacts to be formed (Figure 8A). These interpretations can be confirmed experimentally. According to the definition stated in the CAPPI experiment [37], a contact is said to exist between each pair of residues if at least two heavy atoms are separated by a distance <5 Å. The existence of native contact in each domain cited above is confirmed by residue contact maps of the two hot-spots regions in Figure 8B, which show residues that are closer than the required minimum distance. Additionally, using VMD, we obtained more detailed information about binding interfaces that revealed the C-terminal of A2aR and the N-terminal of D2R, as well as the C-terminal of 5-HT2AR and ICL3 of D2R, adopt a helical conformation at the interface, as observed in Figure 8C and Figure 9C. Residues T85, A84, L81, and A77 of the C-terminal domain of A2aR form a strong interaction with T299, F300, and I303 of the N-terminal of D2R (Figure 8C). Notably, at the C-terminal of 5-HT2AR, residues I341 and V334 further interact with T66, L163, and I159 of ICL3 of D2R (Figure 9C). Another purpose of an MD simulation is often to derive the radius of gyration, which can be used to obtain relevant information about the compactness of a binding domain between two monomers. Figure 10 displays its average over five trajectories for each heterodimer, all of which have reached thermal equilibrium. Finally, it is worth noting that A2aR/D2R hot-spot simulations reached thermal equilibrium after 100 ns of simulation time, whereas 5-HT2AR/D2R took 130 ns, highlighting the difference in structural properties of both complexes at their interfaces.

## 3. Methods

### 3.1. Multiple Sequence Alignment

The protein sequences were retrieved from the UniProt [38] and NCBI [39] websites. For each protein (see Table 1), we downloaded orthologous sequences using *blastp* and retained only sequences with 70–90% similarity with the human protein. These orthologous sequences were then joined by species and aligned using clustal-Ω [40] to generate a concatenated multiple sequence alignment (cMSA) of A2aR/D2R, CB1R/D2R, A2aR/CB1R, 5-HT2AR/D2R, 5-HT2AR/mGluR2, and 5-HT2AR/5-HT4R in 193, 404, 303, 518, 626, and 515 vertebrates, respectively. Only the isoform with the highest sequence similarity to human protein was considered for species with several isoforms. Finally, the resulting cMSA was manually inspected for abnormalities and saved for coevolutionary analysis.

### 3.2. Structure Prior-Assisted Direct Coupling Analysis (SP-DCA)

The pseudo-likelihood maximization DCA (*plmDCA*) is a variant of DCA with higher precision than similar algorithms [41]. The *plmDCA* algorithm requires a multiple sequence alignment (MSA) as input, a table with aligned evolutionary-related amino-acid sequences. Each row of this table is an amino acid coded by one letter of the alphabetical abbreviation for amino acids. The raw output of *plmDCA* generally contains false positives, especially for large proteins. The SP-DCA approach effectively deals with large proteins by integrating secondary structure information of the proteins into the native contacts prediction algorithm. However, the SP-DCA convolves the DCA signal with a Gaussian kernel function built on the secondary structure information of both proteins. Convolved evolutionary coupling score globally depends on the normalized variances a and b of the Gaussian distribution, the total number of amino acids at the interface (*l*), and the evolutionary score of residues of the two proteins as defined in Fongang et al. [4]. The final model illustrating the SP-DCA algorithm is given by Equation (1).

(1) $$Q_{i,j}=\overset{i+l}{\underset{\alpha =i-l}{\sum }}\overset{j+l}{\underset{\beta =j-l}{\sum }}P_{i,j}\exp\left[-\left(a{\left(\alpha -i\right)}^{2}+b{\left(\beta -j\right)}^{2}\right)\;\right]$$

where $P_{i,j}$ is the evolutionary score of residues i and j of the two proteins. Biologically, l is the average length of the peptides involved in the interaction. α and β describe the dependence on the secondary structure of the proteins.

### 3.3. Molecular Dynamic Simulations

We used the crystal structures of D2R (PDB ID: 6CM4) [42], A2AR (PDB ID: 5N2R) [43], and 5-HT2AR (PDB ID: 6A94) [44] to conduct the coarse-grained molecular dynamics simulations. For each complex, A2AR/D2R and 5-HT2AR/D2R, we ran the simulations according to the predicted hot-spots from the SP-DCA analysis. For the A2AR/D2R complex, we performed the simulations using residues from the C-terminal domain of A2AR (C245 to S305) and the N-terminal domain of D2R (N35 to W100), as highlighted in the SP-DCA contact map (Figure 1). For the 5-HT2AR/D2R heterodimer, we performed the simulations using residues from the N-terminal domain of D2R (T144 to R222) and the C-terminal domain of 5-HT2AR (E313 to E401). The initial condition of our MD simulations consists of the two monomers placed in a simulation box at a distance of 20 Å apart from each other at a temperature of 300K with an initial velocity defined using the Boltzmann distribution. We performed the simulations using the transferable force field AWSEM (Associative Memory, Water-mediated, Structure, and Energy Model), a memory-efficient and accurate model for coarse-grained simulation. AWSEM has been used to successfully predict the dynamics of proteins, including calmodulin, a multifunctional calcium-binding protein [34,45]. The Hamiltonian used in the AWSEM force field, defined in Equation (2), is composed of two main terms: physics-based and bioinformatics-based.

(2) $$V_{AWSEM}=V_{backbone}+V_{contact}+V_{burial}+V_{HB}+V_{DH}+V_{FM}$$

The physics-based terms comprise the backbone, contact, burial, hydrogen bond (HB), the Debye-Huckel (DH), and the bioinformatics (FM) terms. The backbone geometry of the protein chains is maintained due to the $V_{backbone}$ term. It includes five terms illustrating the connectivity of the chain: a correct bond around the $C_{\alpha }$ atoms, chirality for the suitable orientations of the $C_{\beta }$ atoms, the excluded volume potential to prevent chain collapse and unphysical entanglements, and the backbone dihedral angles, respectively. The electrostatic effects are considered by using the Debye-Huckel term.

On the other hand, $V_{FM}$ denotes a bioinformatics term that uses the known structure from the protein database (Protein Data Bank) to improve the conformational search of the global minimum. It is based on short fragment sequences named “memory,” as described in the original AWSEM paper [45].

The expression of the Debye-Huckel (DH) potential [46] is given in Equation (3)

(3) $$V_{DH}=K_{Elec}\underset{i<j}{\sum }\frac{q_{i}q_{j}}{\varepsilon_{r}r_{ij}}e^{-r_{ij}/l_{D}}$$

where $q_{i}$ and $q_{j}$ are charges of $C_{\beta }$ atom of each residue i and j; $r_{ij}$ is the distance between them; $\varepsilon_{r}$ refers to the dielectric constant of the media; $K_{Elec}={\left(4\pi \varepsilon_{0}\right)}^{-1}=332.24\;\mathrm{kcal}\cdot {\mathrm{mol}}^{-1}\cdot e^{-2}\;Å$; and $l_{D}$ represents the Debye-Huckel screening length expressed as $l_{D}=\sqrt{\varepsilon_{r}\varepsilon_{0}k_{B}T/2e^{2}I}$, $k_{B}$ is the Boltzmann constant, T is the temperature, e refers to elementary electric charge, and I is the ionic strength of the implicit solvent.

Similar to many other coarse-grained models, AWSEM deals with the compromise between accuracy and computational efficiency. The fewer the explicitly treated atoms (beads) representing each residue, the faster the simulation, and the lower the accuracy. Although AWSEM has been used to successfully predict the structure and dynamics of proteins and complexes, it remains limited in terms of atomistic details. It uses three beads to represent each residue, compared to the average of 20 atoms per residue in all atomistic systems; this leads to a faster simulation time with lower accuracy. On the other hand, these details are often recalled from the short fragment memories of nine residues of length or less used in the AWSEM force field FM term. Furthermore, despite the reduced dimensionality of the system, AWSEM remains applicable to only proteins and/or protein complexes with relatively moderate size, as a result of the longer simulation time required for larger systems.

A molecular dynamics (MD) simulation was carried out on the LAMMPS platform [47], in which the AWSEM potential is implemented [34]. In the simulation protocol, we adopted the periodic boundary conditions of 400 Å on each side of the cubic box. Initial velocities were chosen randomly from the Boltzmann distribution, with the average squared velocity equal to $3K_{B}T/m$. Additionally, we used the canonical ensemble and the Nose-Hoover thermostat to control the temperature, which was fixed at 300K. We carried out 5-replicat simulations for each complex with different initial velocities and an integration time step of 2 fs. The coordinates were recorded every 1000-time steps for further analysis. As validation criteria, we used the radius of gyration, root mean square deviation, probability of native contact formation, and the 2-dimensional protein contact maps. Finally, we visualized the structures using the visual molecular dynamics (VMD) package [48].

## 4. Conclusions

The computational methods to predict the binding interfaces of protein complexes are needed to complement the labor-intensive experimental techniques. Here, we demonstrated that a two-step procedure in which the structure-prior direct coupling analysis (SP-DCA) is combined with a biased molecular dynamic simulation can accurately predict binding interfaces and provide structural details of large protein complexes, including G-protein coupled receptor (GPCRs) heterodimers. In addition, we have applied the procedure to predict the binding interfaces of several GPCRs heterodimers, including the A2aR/D2R, CB1R/D2R, A2aR/CB1R, and the 5HT2AR/D2R. As our predictions all agreed with the experimental data, we extended the methodology to the 5-HT2AR/5-HTR4R heterodimer, given its implication in drug addiction and neurodegenerative pathologies such as Alzheimer’s disease (AD). Furthermore, SP-DCA analysis showed that 5-HT2AR and 5-HTR4R heterodimerize through the C-terminal domain of 5-HT2AR and ICL3 of 5-HT4R. Elucidating the details of GPCR interactions could help accelerate the discovery of druggable sites and improve our knowledge of the etiology of common diseases, including AD.

## Figures and Tables

**Table 2:** Molecular dynamic simulations outputs. For each heterodimer, we provide the closest amino acids after interacting peptides have reached thermal equilibrium. We also provided the final conformation at the interfaces which are all helical.

Complex	MD Output	Main Observation
	A2aR: T85, A84, L81, and A77	
A2aR/D2R	D2R: T299, F300, and I303	Helical conformation
	CB1R: V87, L89	
CB1R/D2R	D2R: S115, M113	Helical conformation
	A2aR: L89, K91	
A2aR/CB1R	CB1R: L174, L176	Helical conformation
	5-HT2AR: I34, and V334	
5-HT2AR/D2R	D2R: T66, L163, and I159	Helical conformation
	5-HT2AR: E6, D45	
5-HT2AR/mGluR2	mGluR2: I125, N106	Helical conformation
	5-HT2AR: T13, L11	
5-HT2AR/5-HTR4R	5-HTR4R: I116, N114	Helical conformation

## Data Availability

The data used in this study are publicly available and the scripts can be downloaded at https://github.com/FongangLab/SP-DCA (last accessed on 2 February 2023).

## Sample Availability

Samples of the compounds are not available from authors.
